# Determination of fatty acid, cholesterol, alpha-tocopherol, and aroma components of traditionally and commercially produced Trabzon butters

**DOI:** 10.1016/j.heliyon.2024.e35656

**Published:** 2024-08-06

**Authors:** Engin Gündoğdu, Musa Beşer

**Affiliations:** Gümüşhane University, Faculty of Engineering and Natural Sciences, Department of Food Engineering, 29100, Gümüşhane, Turkey

**Keywords:** α-Tocopherol, β-carotene, Cholesterol, Fatty acid, Peroxide value, Traditional butter

## Abstract

This study investigated 29 butter samples from Trabzon, Turkey. Cholesterol contents ranged from 134.13 to 325 mg/100 g, α-tocopherol contents ranged from 1.62 to 3.37 mg/100 g, and β-carotene contents ranged from 4.46 to 15.60 μg/g. Fatty acid composition analysis showed variations, with palmitic acid ranging from 26.11 % to 44.25 %, oleic acid from 19.55 % to 29.80 %, and linoleic acid from 1.63 % to 3.04 %. A total of 44 aroma components were identified. Traditional butter samples exhibited differences in aroma components and fatty acid composition compared to commercial butter. Notably, traditional butter had higher concentrations of octanoic and n-decanoic acids than commercial butter. Moreover, some aroma components such as N-butanoic acid 2-ethylhexyl ester, decanoic acid, and pentadecane were found exclusively in traditional butters. Traditional butter showed higher α-tocopherol and β-carotene contents. These findings underscore the distinct chemical profiles of traditional and commercial butter samples, influenced by production methods and possibly geographical origin.

## 1 Introduction

A primary factor influencing consumer butter preferences is the perception of a “natural image.” Consumers often consider dairy products derived from cows that graze on fresh grass as more natural, with an even stronger preference for butter [47].

These quality attributes are influenced by the flora of meadows and pastures, which result in natural differences in taste, aroma, color, quality, and composition of milk and the butter produced from it [33,45]. Additionally, regional climate and production site temperatures contribute to these variations, giving rise to distinct regional butter varieties in Turkey, with notable production in Kars, Erzurum, Urfa, and Diyarbakır, and Trabzon [13].

The distinct flavor and aroma of Trabzon butter are attributed to livestock raised in the Black Sea Region's rich natural habitats. Traditional “yellow butter” from this region is produced from sheep, goat, cow milk, or their mixtures using wooden barrels called külek and churns known as yayık. The cream is collected, churned, and the resulting butter is washed and either consumed fresh or salted for long-term storage [42]. In contrast, commercial butters from Trabzon are produced in factories using locally sourced milk, sometimes supplemented with pasteurized, starter-culture-matured creams from outside the region due to raw material scarcity and quality issues. These commercial butters, often enhanced with colorants like beta-carotene, generally lack the sensory appeal of traditional butters (Private communication).

Variations in quality due to improper processing, inadequate storage, or adulteration with inedible fats, vegetable oils, waste oils, or industrial creams in both industrial and traditionally produced butters can adversely affect consumer acceptance and health, leading to diminished marketability and economic losses [20].

Butter is notable for its rich composition of fatty acids, cholesterol, tocopherol, and aroma components. It contains over 400 different fatty acids [45] with varied biological and nutritional properties essential for human health, along with significant levels of cholesterol and tocopherol, which contribute to its nutritional value and sensory qualities [43]. Cholesterol, a critical component of cell membranes and a precursor for bile acids and steroid hormones, plays a significant role in butter's nutritional profile [29]. Tocopherol, a form of vitamin E, is essential for its antioxidant properties, which help in maintaining butter quality and health benefits [14,25].

The unique aroma and flavor of butter are influenced by its complex lipid composition and the presence of specific volatile compounds [11]. These aroma components are crucial for the sensory appeal of butter, affecting consumer preference and marketability. Recent trends show a growing demand for traditional dairy products, driven by their perceived nutritional and health benefits. Studies on Trabzon butter have primarily focused on its microbiological, chemical, and fatty acid profiles, often using market samples of unknown age and storage history. This study aims to distinguish between traditional and commercial Trabzon butters by evaluating fresh samples directly from producers under controlled conditions.

A comparative analysis of commercial creamy butter, traditional homemade butter, and home-industrial butter revealed that quality is determined by organoleptic parameters, moisture, fat, free fatty acid content, cholesterol, tocopherol levels, and aroma components. Sensory evaluations through buttered pancakes showed commercial creamy butter performed well in all quality parameters, whereas homemade traditional butter excelled organoleptically. In contrast, traditional butters from local markets did not meet the same quality standards [51].

Butter's distinct texture and flavor are largely due to its lipid content, which influences sensory properties like mouthfeel, color, texture, and stability. The specific fatty acid composition affects the physical properties, nutritional value, and overall quality of butter [43]. Cholesterol's critical role in cell membrane structure and function, along with tocopherol's antioxidant properties, underscores their importance in butter’s nutritional profile [29]. Despite historical concerns about the health impacts of saturated fats in butter, recent studies suggest potential bioactive properties against chronic inflammation, highlighting the complex health implications of butter consumption [31].

Hand-made butter is often regarded as an artisan food with superior sensory quality compared to retail brands, based on online opinions and small surveys. However, there are no scientific studies in peer-reviewed literature that compare hand-made butter with industrially produced butter in terms of sensory quality or the chemical compounds affecting flavor and odor. Additionally, there is no official data on hand-made butter consumption or sales. Many farm shops promote their hand-made butter online, using milk from their own herds and traditional methods. However, “traditional” is rarely defined, making it difficult to assess its impact on the butter's shelf-life. This highlights the need for detailed studies on the production processes and quality characteristics of hand-made versus industrial butter [15].

As consumers increasingly seek foods with traditional character and local origins, the demand for products like Trabzon butter has risen. To our knowledge there is a few study on Trabzon butters [4,42].

Studies have shown that butters sold in Trabzon's local markets are often considered traditional. However, there are reports that some of them purchased from supermarkets are being sold in these markets under the guise of traditional butter (Private communication). Therefore, accepting all market-sold ones as traditional does not accurately represent Trabzon butter. Additionally, some labeled as Trabzon butter are made from cream sourced from commercial enterprises or from different districts of Trabzon due to insufficient local cream supplies, which negatively affects butter quality. Thus, special efforts were made to obtain samples made from the milk of cows fed on local grasses, which is considered traditional.

Moreover, as to our best knowledge, there are no existing studies analyzing cholesterol, alpha-tocopherol, and beta-carotene in Trabzon butters. Therefore, the main objective of the current study is to investigate the quality differences between traditional and commercial Trabzon butters by focusing on their cholesterol, tocopherol, and aroma components to better understand their unique characteristics and ensure authentic products reach the market.

## 2 Materials and methods

### 2.1 Material

Butter samples were collected from various districts in Trabzon, a region known for its livestock farming. We classified the butter samples obtained directly from local producers as “traditional” and those acquired from facilities as “commercial.” A total of 25 traditional butter samples were collected from the districts of Akçaabat, Araklı, Beşikdüzü, Çaykara, Hayrat, Maçka, Sürmene, Şalpazarı, Tonya, Vakfıkebir, and Yomra. Additionally, five commercial butter samples were obtained from facilities located in Vakfıkebir, Tonya, and Sürmene. These facilities were selected because they have a production capacity of over three tons of Trabzon butter daily. The samples were collected in November 2022 in sterile 150 mL plastic sample containers, with two containers per sample. They were transported to the laboratory under cold conditions and stored at 4 °C. Analyses for pH, acidity, % dry matter, % fat, and % salt were conducted at the Kahvaltı Dünyası Laboratories. Oxidation tests (PV and TBA numbers), color, fatty acids, aroma compounds, cholesterol, tocopherol, beta-carotene, and were performed at the Food Engineering Department laboratories of Gümüşhane University. In order to prevent oxidation, the samples for the determination of fatty acids, volatile aroma compounds, cholesterol, tocopherol, and beta-carotene were stored in a deep freezer at −18 °C until they get analyzed.

### 2.2 Methods

#### 2.2.1 Physicochemical properties of butters

The quality parameters of the butter samples were evaluated through various analytical tests. pH values were measured using a pH meter (Hanna Instruments, pH 211, Romania) [35], while acidity percentage was determined by titrating 5 g of each sample in a 250 mL Erlenmeyer flask with a mixture of alcohol, ether, and 2 % phenolphthalein, then neutralizing with 1/10 N sodium hydroxide [35]. Dry matter content was assessed by weighing 3–4 g of each sample into pre-cooled containers, drying in an oven at 100–102 °C until constant weight, and calculating the percentage [28]. Fat content was determined using the Gerber butyrometer method, where 5 g of butter was mixed with water, sulfuric acid, and amyl alcohol, centrifuged, and the fat content recorded [28]. Salt content was measured by shaking 10 g of sample with hot distilled water, diluting, titrating with 0.1 N AgNO3 [28].

$$\text{Salt}\;\%=\frac{\text{Volume}\;\text{of}\;0.1\;N\;\text{AgNO}3\;\text{used}\;\left(\text{mL}\right)\times 0.0058}{1\;g\;\left(\text{butter}\right)}x\;100$$

where 0.00585 g represents the amount of AgNO₃ solution that reacts with 1 mL of 0.1 N NaCl solution.

#### 2.2.2 Peroxide value

Two grams of butter sample was weighed into a 50 mL Erlenmeyer flask with a ground-glass stopper, and 15 mL of an acetic acid-chloroform mixture was added. After the oil had completely dissolved, 0.5 mL of saturated potassium iodide solution was added and shaken vigorously for 1 min. The mixture was kept in the dark for 5 min, then mixed with 30 mL of distilled water and 1 mL of starch solution, and titrated with 0.002 N sodium thiosulfate, calculating from the following formula [35].

$$PV=\frac{\left(V1-V0\right)XN}{M}$$

where V1 is the amount of Na_2_S_2_O_3_ used for titration (mL), V0 is the amount of Na_2_S_2_O_3_ used for the blank (mL), N is the normality of Na_2_S_2_O_3_ and M is the amount of sample (g). PV are expressed as milliequivalents (meq) of active oxygen per kg of butter.

#### 2.2.3 Thiobarbituric acid number

In a 10 mL glass flask, 0.08 g of butter was weighed and completed to volume with n-butanol. Subsequently, 5 mL of this sample and 5 mL of TBA solution were added to a tube. The mixture was then heated at 90 °C for 120 min with the lid closed. A blank was prepared in the same manner. After cooling, the absorbance was read at 530 nm using a UV-VIS spectrophotometer (Shimadzu, UV-1208). The TBA value of the butter sample was determined using the following formula, based on the measured absorbance (optical density) values [1].

$$TBA\;value\;\left(mg\frac{MAD}{kg}fat\right)=\frac{\left(A-B\right)}{m}$$

where, A: Absorbance of the sample, B: Absorbance of the blank,m: Mass of the sample.

#### 2.2.4 Color values

The color of the butter was measured using a Konica Minolta CR-400 ChromaMeter (Konica Minolta, Tokyo, Japan). The parameters used for color evaluation included brightness (L*), red-green parameter (a*), and yellow-blue parameter (b*).

#### 2.2.5 Determination of cholesterol content

For determining the cholesterol content, a cholesterol stock solution (2 mg/mL) was first prepared. Specifically, 20 mg of cholesterol standard was dissolved in hexane in a 10 mL volumetric flask. Solutions ranging from 10 to 80 mg/mL were then prepared by appropriately diluting the stock solution with hexane. For the analysis, 1 g of butter was weighed and 5 mL of methanolic KOH solution was added. The tube was tightly capped and vortexed for 15 s. The tubes were then placed in an 80 °C water bath for 15 min, vortexing for 10 s every 5 min. After cooling the tube with tap water, 1 mL of water and 5 mL of hexane were added, and the mixture was vigorously vortexed for 1 min, followed by centrifugation at 2000×*g* for 1 min. The upper phase was collected, and the cholesterol content was determined using GC-MS/FID [18]. For this analysis, a capillary column coated with 25 m × 0.32 mm x 0.17 μm 5 % phenyl-methyl - 95 % dimethyl polysiloxane was used for the GC analysis. Helium was employed as the carrier gas. The injector temperature was set at 250 °C, while the detector (FID) temperature was maintained at 300 °C. The column temperature was also held at 300 °C. An injection volume of 1 μL with a split ratio of 1:20 was utilized. A 1 μL injection from each standard working solution was made, and a calibration curve was constructed using the areas obtained from the GC-MS/FID instrument's FID detector and the corresponding concentrations. The slope, intercept, and least squares fit of the standard curve were calculated. Using the calibration curve, the cholesterol content in the samples was calculated as mg/100 g.

#### 2.2.6 Determination of alpha-tocopherol and beta-carotene contents

A spectrophotometric method was used for this purpose. For the calibration curve, 25.0 mg of β-carotene was transferred to a 25 mL volumetric flask and dissolved in ethanol at 40 °C containing 0.025 % BHA. This solution was then diluted with PBS to obtain concentrations ranging from 0.005 to 0.1 mg/mL. The same procedures were applied for alpha-tocopherol.

For the determination of alpha-tocopherol, 1 g of butter sample was dissolved in ethanol at 40 °C in a 25 mL volumetric flask. From this solution, 1 mL was transferred to a 10 mL volumetric flask and diluted with PBS. Subsequently, 1 mL of this solution was transferred to a tube, and 5 mL of a hexane/ethanol (3:1) mixture was added. The absorbance values were measured at 450 nm, and the concentration was calculated using the standard curve.

For beta-carotene, the same procedures were followed, but 2 g of butter sample was used. From the tubes, 3 mL of sample was taken, and 5 mL of a hexane/ethanol (3:1) mixture was added. The absorbance value was measured at 260 nm. The beta-carotene content was calculated from the slope of the standard curve [30].

#### 2.2.7 Determination of fatty acid composition

The fatty acid composition was determined according to the TS EN ISO 12966-4 method (2015). Fatty acid methyl esters (FAMEs) were prepared by adding 5 mL of n-hexane and 100 μL of 2 N KOH (in methanol) to the sample. The FAMEs were then injected into a GC-MS/FID detector (Agilent 5975, Agilent Technologies Inc., USA) equipped with a column. The analysis was performed using a RESTEK Rtx-2330 column (fused silica), 30 mm × 0.25 mm x 0.2 μm (Cat. No 10723, Serial No 1081624). The flow rate was set at 1.8761 mL/min, with a pressure of 21.231 psi, and the flow condition was maintained at constant pressure. The GC-MS/FID settings were as follows: Detector column: 30 m × 0.25 mm ID, 0.2 μm HP-5MS; Oven: 50 °C, 25 °C/min to 200 °C, hold for 1 min; 3 °C/min to 230 °C; Carrier gas: Helium, constant pressure Injection: 250 °C; Detector: MS, 230 °C; Split ratio: 1.20; Injection volume: 1 μL; H2: 40 mL/min; Dry air: 450 mL/min. In fatty acid analysis, the Supelco 37 Component FAME Mix was used to determine retention times. The results were calculated as percentage area using the area normalization method from the chromatograms obtained at the end of the injection. The percentage of fatty acid was calculated using the formula:

$$\text{Fatty}\;\text{acid},\%=\left(\frac{A\left(\text{Fatty}\;\text{acid}\;\text{methyl}\;\text{ester}\right)}{A(\text{Total}\;\text{fatty}\;\text{acid}\;\text{methyl}\;\text{ester}}\right)x100$$

where A is the integrated peak area.

The fatty acid content was expressed as a percentage of the total fatty acid content. Fatty acid composition was determined in 10 samples that complied with the Turkish Food Codex (No: 2005/19) regarding dry matter, fat, and water content.

#### 2.2.8 Determination of volatile aroma compounds

The extraction of aroma compounds was conducted using an in-house method with a 75 μm Carboxen-PDMS SPME fiber. A 2.5 g homogenized butter sample was weighed into a 20 mL SPME vial and diluted with a saturated NaCl solution in a 1:1 ratio. The vial was tightly sealed and thoroughly mixed using a tube shaker to ensure complete dissolution, then incubated at 70 °C in a water bath for 10 min. A 75 μm Carboxen-PDMS SPME fiber, pre-conditioned at 250 °C, was placed in the vial and maintained at 70 °C for 40 min. Following this, the fiber was transferred to the GC-MS injection port and held at 250 °C for 15 min. The analysis was performed using an Agilent 5975 GC-MS detector. The column used was 30 m × 0.25 mm ID, 0.2 μm HP-5MS. The oven temperature program started at 50 °C and increased at a rate of 4 °C/min to 260 °C, where it was held for 15 min. Helium was used as the carrier gas at a constant flow rate of 1.2 mL/min. The identification of aroma compounds was conducted by comparing the retention index (RI) values of each volatile compound in the butter samples with literature values obtained under the same working conditions (http://webbook.nist.gov/chemistry). Volatile aroma compounds were determined in 10 samples that complied with with the Turkish Food Codex (No: 2005/19) regarding dry matter, fat, and water content.

#### 2.2.9 Statistical analysis

All analyses were made dublicate and results were given as mean ± standard deviation (SD). PCA were performed to evaluate the fatty acid composition using the software package (XLSTAT Addinsoft SARL 2019).

## 3 Results and discussion

While physical and chemical analyses were conducted on all samples, those with a fat content of 80 % or higher and a water content of 16 % or lower, as per the Turkish Food Codex (No: 2005/19). Eight samples obtained directly from producers and the two from facilities met the criteria established by Turkish Food Codex (No: 2005/19). In total, 10 samples were analyzed for cholesterol, beta-carotene, alpha-tocopherol, fatty acids, and aroma components.

### 3.1 Physical and chemical properties of butter samples

Table 1 presents the results of the pH, lactic acid, dry matter, fat, and salt values of the butter samples.

**Table 1:** PH, acidity, drymatter, fat, moisture and salt contents of butter samples.

**Samples**^a^	pH	**Acidity (% LA)**	**Drymatter (%)**	**Fat (%)**	**Moisture content (%)**	**Salt (%)**
1	4.45	0.28	73.25 ± 0.78	70.72 ± 1.02	26.75 ± 0.78	0.07
2	4.82	0.21	83.58 ± 0.59	82.03 ± 0.75	16.42 ± 0.59	0.07
3	4.42	0.57	68.48 ± 0.46	65.50 ± 0.70	31.53 ± 0.46	1.24
4	4.53	0.28	71.04 ± 0.23	68.30 ± 0.43	28.96 ± 0.23	0.09
5	4.38	0.25	82.50 ± 0.99	80.84 ± 1.19	17.50 ± 0.99	0.09
6	4.41	0.60	85.21 ± 2.25	83.81 ± 2.55	14.79 ± 2.25	0.07
7	4.32	0.18	79.84 ± 1.08	77.93 ± 1.31	20.17 ± 1.08	0.77
8	3.90	0.14	84.36 ± 0.23	82.89 ± 0.16	15.64 ± 0.23	0.07
9	4.12	0.28	82.40 ± 0.85	80.74 ± 1.04	17.60 ± 0.85	0.89
10	4.86	0.28	82.60 ± 1.13	80.95 ± 1.35	17.40 ± 1.13	0.28
11	4.89	0.39	77.30 ± 0.71	75.15 ± 0.92	22.70 ± 0.71	0.70
12	4.91	0.14	83.54 ± 0.76	81.99 ± 0.73	16.46 ± 0.76	0.37
13	4.48	0.25	82.78 ± 0.54	81.16 ± 0.48	17.22 ± 0.54	0.05
14	4.56	0.32	83.94 ± 0.19	82.42 ± 0.11	16.06 ± 0.19	0.19
15	5.08	0.28	78.65 ± 1.34	76.63 ± 1.60	21.35 ± 1.34	0.07
16	4.43	0.25	85.04 ± 0.05	83.63 ± 0.04	14.96 ± 0.05	1.22
17	4.30	0.21	86.15 ± 0.36	84.84 ± 0.48	13.85 ± 0.36	0.07
18	5.14	0.14	86.78 ± 0.60	85.52 ± 0.74	13.23 ± 0.60	0.07
19	4.30	0.11	84.79 ± 0.27	83.36 ± 0.20	15.21 ± 0.27	0.16
20	4.41	0.18	87.23 ± 0.04	86.02 ± 0.03	12.77 ± 0.04	0.07
21	4.02	0.25	83.13 ± 1.23	81.53 ± 1.46	16.87 ± 1.23	0.09
22	4.45	0.32	82.65 ± 0.08	81.02 ± 0.02	17.35 ± 0.08	0.09
23	4.78	0.32	84.77 ± 0.33	83.33 ± 0.46	15.24 ± 0.33	0.09
24	5.20	0.21	83.95 ± 1.76	82.43 ± 2.03	16.05 ± 1.76	0.26
**Mean**	**4.55**	**0.27**	**81.83**	**80.11**	**18.17**	**0.30**
25	5.43	0.32	83.33 ± 1.52	81.75 ± 1.76	16.67 ± 1.52	0.09
26	4.73	0.28	84.77 ± 0.33	83.33 ± 0.46	15.24 ± 0.33	0.30
27	4.51	0.28	84.21 ± 0.83	82.72 ± 1.01	15.79 ± 0.83	0.02
28	4.70	0.28	83.13 ± 1.23	81.53 ± 1.46	16.87 ± 1.23	0.40
29	4.64	0.32	82.63 ± 0.53	80.98 ± 0.69	17.37 ± 0.53	0.07
**Mean**	**4.80**	**0.30**	**83.61**	**82.06**	**16.39**	**0.18**
Min	3.9	0.11	68.48 ± 0.46	65.50 ± 0.70	12.77 ± 0.04	0.02
Max	5.43	0.6	87.23 ± 0.04	86.02 ± 0.03	31.53 ± 0.46	1.24
Grand Mean	4.59	0.27	82.14 ± 4.38	80.45 ± 4.79	17.86 ± 4.38	0.28

a 1–24: traditional butters; 25–29: Commercial butters.

**Table 2:** PV and TBA values of butters.

Samples^a^	PV (meq O_2_/kg)	TBA (mg MAD/kg)
1	0.359 ± 0.03	0.228 ± 8.61
2	1.396 ± 0.20	0.252 ± 1.24
3	0.387 ± 0.00	0.212 ± 1.22
4	0.464 ± 0.03	0.289 ± 1.73
5	0.660 ± 0.05	0.183 ± 0.58
6	0.338 ± 0.03	0.161 ± 0.64
7	1.527 ± 0.30	0.275 ± 7.34
8	0.303 ± 0.30	0.260 ± 6.68
9	0.232 ± 0.03	0.227 ± 9.27
10	0.653 ± 0.00	0.252 ± 4.69
11	2.109 ± 0.38	0.355 ± 2.48
12	0.322 ± 0.16	0.154 ± 1.87
13	0.000 ± 0.00	0.237 ± 13.21
14	1.377 ± 0.02	0.191 ± 2.30
15	0.270 ± 0.03	0.110 ± 2.63
16	0.136 ± 0.03	0.292 ± 4.63
17	0.328 ± 0.04	0.155 ± 4.42
18	0.259 ± 0.01	0.175 ± 2.46
19	0.000 ± 0.00	0.213 ± 7.47
20	0.000 ± 0.00	0.120 ± 3.79
21	0.666 ± 0.00	0.153 ± 7.78
22	0.544 ± 0.03	0.367 ± 19.71
23	0.130 ± 0.00	0.325 ± 1.25
24	0.140 ± 0.08	0.185 ± 0.64
**Mean**	**0.53**	**0.22**
25	0.140 ± 0.08	0.185 ± 0.64
26	0.359 ± 0.03	0.150 ± 7.53
27	1.396 ± 0.02	0.117 ± 1.52
28	0.387 ± 0.03	0.268 ± 11.47
29	0.464 ± 0.00	0.123 ± 12.30
**Mean**	**0.55**	**0.17**
Min	0.130 ± 0.00	0.110 ± 2.63
Max	2.109 ± 0.38	0.367 ± 19.71
Grand Mean	0.484 ± 0.50	0.214 ± 0.07

a 1–24: traditional butters; 25–29: Commercial butters.

In terms of dry matter and fat content, 20 % of the samples fell below the standards of the Turkish Food Codex (No: 2005/19). However, all samples were compliant in terms of salt content. The salt values of butter samples ranged from 0.07 % to 1.22 % (Table 1). The pH values of the samples ranged from 3.90 to 5.43. The Turkish Food Codex (No: 2005/19) does not specify any criteria for pH values. However, commonly available types of butter in the market include immature cream butter, matured cream butter, pasteurized cream butter, unsalted butter, salted butter, sour cream butter, sweet cream butter, cold storage butter, fresh cream butter, peanut butter, milk fat, cocoa butter, and creamy butter [26]. Commercial butters exhibit a higher mean pH (4.80) compared to traditional butters (4.55), possibly due to different cultures or processing methods resulting in less acidic conditions. Acidity is slightly higher in commercial butters (0.30 %) than in traditional butters (0.27 %), likely due to controlled fermentation. Commercial butters also have higher dry matter content (83.61 %) and fat content (82.06 %) compared to traditional butters (81.83 % and 80.11 %, respectively), reflecting standardized production processes. Traditional butters contain more moisture (18.17 %) than commercial butters (16.39 %) due to less rigorous dehydration. Additionally, salt content is higher in traditional butters (0.30 %) compared to commercial butters (0.18 %). The results have shown that samples, other than those given in Table 4, fall outside the specifications of the Turkish Food Codex (No: 2005/19).

**Table 3:** Color values of butters.

Samples^a^	*L*^a^	*a*^a^	*b*^a^
1	88.42 ± 0.76	−5.09 ± 0.42	32.17 ± 0.31
2	82.60 ± 0.19	−4.87 ± 0.16	37.07 ± 0.41
3	87.45 ± 1.88	−4.77 ± 0.11	30.97 ± 1.25
4	83.37 ± 0.68	−5.22 ± 0.17	32.76 ± 0.46
5	83.47 ± 0.74	−5.28 ± 0.13	31.46 ± 0.54
6	84.90 ± 1.81	−4.82 ± 0.11	29.51 ± 0.60
7	79.67 ± 0.14	−5.42 ± 0.12	36.53 ± 0.49
8	81.79 ± 0.42	−5.33 ± 0.05	26.36 ± 0.11
9	80.95 ± 1.21	−5.96 ± 0.10	38.00 ± 0.17
10	86.22 ± 0.99	−3.71 ± 0.22	42.70 ± 0.53
11	85.73 ± 1.51	−4.34 ± 0.26	34.29 ± 0.97
12	82.13 ± 0.31	−6.27 ± 0.12	46.43 ± 0.87
13	86.12 ± 0.98	−4.37 ± 0.03	28.88 ± 0.52
14	81.80 ± 0.18	−4.20 ± 0.23	18.70 ± 2.29
15	84.97 ± 1.65	−3.60 ± 2.68	21.34 ± 0.31
16	78.35 ± 1.09	−5.50 ± 0.24	42.11 ± 1.43
17	83.94 ± 0.38	−5.78 ± 0.18	38.13 ± 0.30
18	81.64 ± 0.40	−5.67 ± 0.05	33.75 ± 0.44
19	82.82 ± 0.83	−4.79 ± 0.15	33.10 ± 0.90
20	83.74 ± 1.10	−5.36 ± 0.16	29.68 ± 1.62
21	86.17 ± 1.06	−5.73 ± 0.20	39.64 ± 0.29
22	84.43 ± 1.12	−4.50 ± 0.15	32.78 ± 1.61
23	81.45 ± 0.81	−6.19 ± 0.11	39.18 ± 0.90
24	80.66 ± 0.98	−4.50 ± 0.16	41.83 ± 0.56
**Mean**	**83.45**	**−5.05**	**34.06**
25	85.35 ± 1.22	−4.85 ± 0.06	37.74 ± 0.54
26	83.30 ± 0.53	−4.93 ± 0.26	34.77 ± 0.71
27	84.22 ± 2.49	−5.36 ± 0.21	25.94 ± 2.24
28	82.27 ± 0.68	−3.98 ± 0.17	36.14 ± 0.33
29	86.54 ± 1.46	−5.93 ± 0.18	40.34 ± 0.87
**Mean**	**84.34**	**−5.01**	**34.99**
Min	78.35 ± 1.09	−3.60 ± 2.68	18.70 ± 2.29
Max	87.45 ± 1.88	−6.27 ± 0.12	46.43 ± 0.87
Mean	83.60 ± 2.34	−5.05 ± 0.70	34.22 ± 6.24

a 1–24: Traditional butters; 25–29: Commercial butters.

**Table 4:** The cholesterol, α-tocoferol, and β-carotene contents of butters.

Samples^a^	Cholesterol (mg/100 g)	α-tocoferol (mg/100 g)	β-carotene (μg/g)
6	134.13 **±** 11.35	1.88 **±** 0.28	5.98 **±** 0.15
8	252.80 **±** 12.37	2.86 **±** 0.18	4.46 **±** 0.46
16	248.08 **±** 0.33	1.95 **±** 0.37	7.12 **±** 1.92
17	144.37 **±** 18.49	2.47 **±** 0.37	8.70 **±** 2.46
18	272.40 **±** 10.84	3.37 **±** 0.37	7.77 **±** 1.31
19	244.57 **±** 1.90	1.88 **±** 0.09	6.25 **±** 0.38
20	268.18 **±** 7.32	2.99 **±** 0.55	13.75 **±** 0.85
23	325.81 **±** 1.08	2.27 **±** 0.64	15.60 **±** 0.69
**Mean**	**236.29**	**2.46**	**8.70**
26	288.90 **±** 6.97	2.73 **±** 0.00	8.37 **±** 0.61
27	275.77 **±** 7.08	1.62 **±** 0.46	4.95 **±** 0.23
**Mean**	**282.34**	**2.18**	**6.66**
**Min**	134.13 **±** 11.35	1.62 **±** 0.46	4.46 **±** 0.46
**Max**	325.81 **±** 1.08	3.37 **±** 0.37	15.60 **±** 0.69
**Mean**	245.50 ± 7.77	2.40 ± 0.33	8.29 ± 0.91

a 6.8.16.17.18.19.20.23–23: Traditonal butters; 26.27: Commercial butters.

Moisture and dry matter ratio are factors affecting the characteristics of butter. Butter with a high dry matter content containing relatively large crystals (>5 μm) exhibits a harder, more brittle, and granular structure compared to butter containing small crystals. Butter with low dry matter content does not form large crystals with fat, resulting in a greasy product. Meanwhile, butter produced from phase inversion of highly concentrated creams forms an extremely hard structure with poor water distribution [43]. Out of the 29 analyzed samples, 10 samples complied with the 16 % moisture content requirement according to the Turkish Food Codex (2005/19) while the remaining samples exhibited higher moisture content varying from 16.05 % to 31.52 %.

Akgül et al. [3] analyzed the butter produced in Trabzon, identifying physical, microbiological, and fatty acid compositions. They found that the fat and water contents exceeded the limits while the salt content was within regulatory standards set by the Turkish Food Codex (No: 2005/19), which is in agreement with our findings. Higher moisture content in traditional butter is likely due to differences in processing equipment and conditions used to prepare butter. In traditional methods, butter granules are formed in fermented milk with higher moisture content than raw materials (cream) used to produce industrial butter. Additionally, in butter with high dry matter, especially due to proteins in the granules, strong bonding of proteins with water surrounding the butter granules prevents water from leaking out during kneading [41]. As seen in Table 1, traditional butter samples have relatively higher moisture content (12.77–31.53) than those of (15.24–17.37) commercial ones. Similarly Ghasemloy Incheh et al. [19] attributed the high moisture content in traditional butter to the traditional butter-making method and the use of milk with higher moisture content than in the industrial preparation protocol, leading to the use of water as a fat substitute in the butter formulation.

Although numerous studies on butter exist outside of Turkey, there are only a limited number of studies on Trabzon butter within the country. Therefore, we believe our findings may contribute to the literature in this area.

### 3.2 PV and TBA values of butter samples

Butter is a dairy product made from sweet cream or sour cream. It is a perishable food that can spoil due to chemical changes during storage. Souring is a significant issue that leads to aroma deterioration and a reduction in the nutritional quality of butter. This problem arises from the lipolysis and oxidation of fatty acids, as determined by the analysis of peroxide value (PV) and thiobarbituric acid (TBA) parameters [46]. Middaugh et al. [36] utilized a total of 30 samples of butter to determine the chemical quality or fatty acid profile of traditional butter in Zanjan, Iran. They found that the moisture content of all butter samples, as well as the peroxide value (PV), did not fall within the standard range. The authors suggested that various factors affecting the composition of milk, such as traditional production methods, environmental conditions, animal species, lactation period, and geographical location, could influence the chemical composition of butter samples. Therefore, they recommended ensuring hygienic conditions during the production process and maintaining the cold chain (4 ± 1 °C) until consumption to maintain the chemical quality of butter samples. PV and TBA results of butter samples in our study are given in Table 2. As seen, PV was not detected in three samples, while in others, it ranged from a minimum of 0.30 to a maximum of 2.109 meq O_2_/kg fat. When evaluated in terms of TBA values, they ranged from 0.110 to 0.367.

In a study conducted in Azerbaijan, traditional butter samples varied between 0 and 7.33 meq O_2_/kg, deviating from the standard value of 1.7 meq O_2_/kg (58.3 %) [19]. Sevmiş et al. [50] determined peroxide values in butter from the Hakkâri market to be between 1.15 and 6.69 meq O2/kg fat, and TBA values between 0.12 and 0.36 mg MDA/kg butter. In a study, peroxide values of traditional butter were found to be between 1.2 and 7.4 meq O_2_/kg fat [17]. Bayır et al. [8] determined peroxide values to be between 0.60 and 4.67 meq O_2_/kg fat. Çavdar [12] determined PD at 3.25 meq O_2_/kg fat and TBA value at 0.09 mg MDA/kg fat in butter samples collected from the market. Akgül et al. [4] found the peroxide values from 0.00 to 6.84 mEq O_2_/kg for Trabzon butters. The Turkish Food Codex (2005/19) does not provide data on PV (Peroxide Value) and TBA (Thiobarbituric Acid) values. However, Altun et al. [6] stated that PV values above 3 mEq O2/kg indicate oxidative rancidity. From this perspective, all samples were found to have PV values below this specified threshold. The TBA values were higher in traditional butters, which is thought to be due to the higher beta-carotene content in traditional butters (Table 4) and the oxidation of beta-carotene [10].

### 3.3 Color values of butter samples

The natural yellow color of butter primarily originates from the carotene (provitamin A) present in the milk fat and, along with the characteristic microstructure of well-processed butter, gives it a distinctive rich matte surface appearance. A perfect butter slice cuts cleanly and does not appear greasy or shiny [24]. The color of a dairy product can be indicative of physicochemical changes. In this context, color changes occurring especially during storage are significant quality criteria, and therefore instrumental color analysis is commonly used to determine color changes in dairy products during storage. Additionally, the colors of foods need to be analyzed extremely sensitively. Due to the subjective perception of color and the variability from observer to observer, color measurement devices are generally used for reliable and objective color determination [37]. The color values of butter samples are provided in Table 3. Among the color values, the L* value ranged from 78.35 to 88.42, the a × value from −3.60 to −6.19 and b × value from 18.80 to 46.43 in the traditional samples. There are many factors that affect the color parameters, such as the level of fat globule aggregation, which reduces brightness (L*) if it is too high and increases the values of the a × and b × coordinates. Furthermore, the color and size of the fat globules in the initial cream, or the presence or absence of salt, affect the color parameters of the resulting butters [55]. In commercial butter samples, these values varied between 82.27 and 86.54 for L*, −3.98 and −5.93 for a*, and 25.94 and 40.34 for b*. In a study, the L* values were measured between 91.70 and 94.85, the a × values ranged from −6.62 to 7.29, and the b × values were between 19.25 and 33.05 [43].

In a study, it was statistically determined that the dry matter, fat, salt content, and peroxide number of butter were correlated with its color values. Additionally, the Polenske number and fatty acid content were associated with the color values. The authors suggested that fatty acids might influence the color of butter, but they noted that further studies are needed to confirm these findings [2]. Nowadays, consumers tend to be more educated and concerned about the health, fitness properties, and food safety of a food product. When people purchase dairy products, the first thing they can look at is appearance. This for, the color of a product plays a noteworthy role in food choice. Overall, it is well-known that color is one of the main factors in consumer preference. Typically, customers associate color with freshness, ripeness, attractiveness, and taste [38].

### 3.4 Cholesterol, α-tocopherol, and β-carotene contents of butter samples

**Fig. 1:**
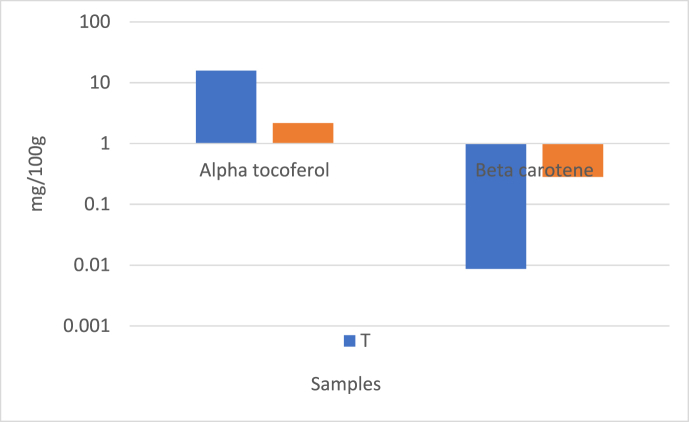

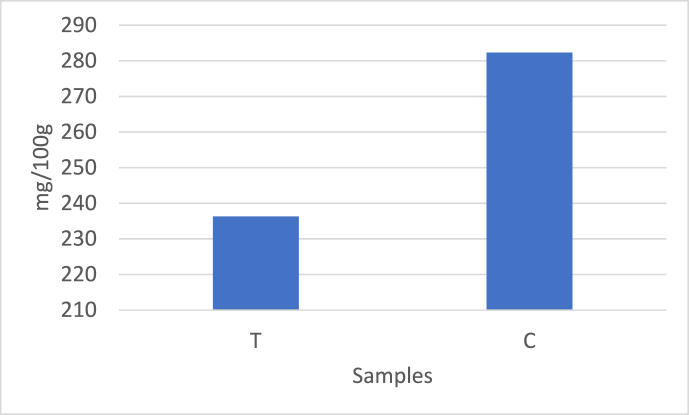
a Alpha tocoferol and beta carotene contents of butter samples. T: Traditional, C: Comercial, b Cholesterol contents of butter samples. T: Traditional, C: Commercial

β-carotene serves as a precursor for vitamin A and is converted to it in the intestines and liver [7]. In recent years, there has been considerable interest in the content of tocopherols and β-carotene due to their ability to prevent the oxidation of milk fat. These compounds are largely influenced by the diet, being particularly abundant in spring and summer months due to the consumption of vitamin-rich fresh forage. Moreover, it has been noted that the content of β-carotene varies according to breeds, with Jersey breeds exhibiting higher levels of β-carotene in milk fat compared to other breeds [22]. The α-tocopherol, β-carotene, and cholesterol content of all butter samples are provided in Table 4. Additionally, the α-tocopherol and β-carotene content of traditional and commercial butters are illustrated in Fig. 1a, while the cholesterol content is shown in Fig. 1b. As observed in Table 4, α-tocopherol contents ranged from 1.62 to 3.37 mg/100g, and β-carotene contents ranged from 4.46 to 15.60 μg/g. Furthermore, traditional butters have higher levels of α-tocopherol and β-carotene compared to commercial ones, as seen in Fig. 1a.

Except for one sample of traditional butter, lower levels of cholesterol and α-tocopherol were found in industrial butter samples, while higher levels of β-carotene were detected in industrial butter samples. This suggests the potential use of β-carotene in industrial butter production.

The secretion of α-tocopherol and β-carotene has been observed to be limited on a daily basis, with the secretion amount reaching its highest level at 29 weeks, although it is initially low at the beginning of lactation. Considering the disproportionate increase in milk yield, it is suggested that the contents of α-tocopherol and β-carotene may be diluted due to limited [22]. Fresh and green plants have been reported to have higher tocopherol and carotenoid contents compared to dried and stored fodder [23].

Cholesterol is an organic substance found in human and animal tissues and cells, playing a significant role in processes such as the synthesis of vitamin D, as well as the utilization of calcium and phosphorus. It is also a crucial component of nerve cells and serves as a precursor for bile acids and sex hormones synthesis [49]. The cholesterol contents in the current study were found to ranged from 134.13 to 325 mg/100g (Table 4). At the same time the mean of traditional and commercial butters were given in Fig. 1b. As seen Fig. 1b, cholesterol was found higher in the commercial butters when compared to the traditional ones. Cholesterol contents in cream and butter are reported to be 190–378 mg/100g and 237–309 mg/100g of fat [39]. The cholesterol contents of butter samples collected from the Hakkari market ranged from 240.64 to 420.64 mg/100 g of fat [50]. The consumption of butter and other high-fat dairy products has decreased in recent years due to the negative perception of dietary fats. This perception is often associated with the belief that cholesterol and saturated fatty acids elevate plasma cholesterol levels and increase the risk of developing coronary heart disease [52]. In a study evaluating the composition of cow's milk butter produced in the Azores archipelago, cholesterol contents (136–143 mg/100 g), β-carotene (0.12–0.17 mg/100 g), and α-tocopherol (1.40–2.20 mg/100 g) were determined. The study also reported that all brands received high scores in terms of appearance, consistency, and flavor, indicating a potentially healthier fat content in Azores butter produced from pasture-based cow's milk, which could be encouraged for product differentiation due to its “natural appearance” [52]. A study revealed that probiotic bacteria have cholesterol-lowering effects in both cream and butter [5]. The study find that no differences in cholesterol content were observed across different seasons or between conventionally and continuously manufactured butter (p = 0.05) [27].

### 3.5 Fatty acid composition

**Fig. 2:**
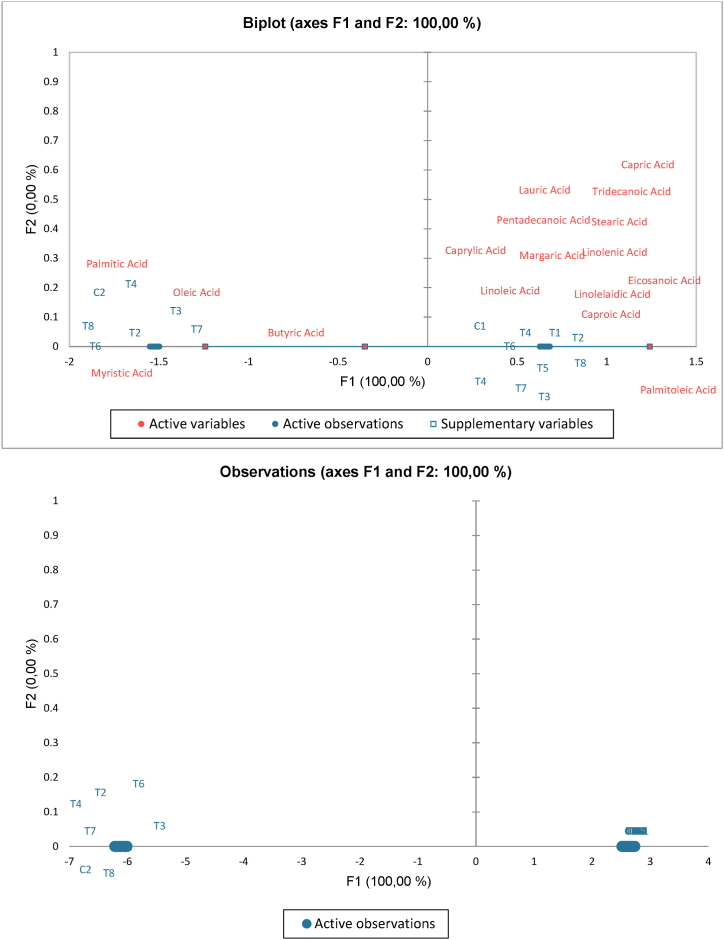
PCA analysis of butter samples.

Milk fat is one of the most complex fats, consisting of approximately 98 g/100 g triglycerides. It primarily contains saturated (66 g/100 g), as well as monounsaturated (38 g/100 g), and polyunsaturated (4 g/100 g) fatty acids (FAs) [48]. The physical properties of milk fat are influenced by the structure of triacylglycerols (TAGs), i.e., the distribution of FAs within TAGs. Milk fat TAGs consist of 26–54 carbon atoms, with TAGs containing 36–40 and 48–52 carbon atoms being the most abundant in milk fat. The distribution of FAs in TAG molecules determines the melting temperature of low, medium, and high melting point TAG fractions in milk fat. The melting behavior of milk fat TAGs affects the rheological properties of butter [53]. In our study, the fatty acid composition of butter samples is presented in Table 5 as percentage values. Additionally, PCA analysis was applied to identify the differences in fatty acids between traditional and commercial butters, as shown in Fig. 2. As shown in Fig. 2 the predominant fatty acids are displayed on the left and right sides of the graph. On the right side, traditional butters are dominated by omega fatty acids such as linoleic, linolenic, and eicosanoic acids. This is thought to be due to the richness of the local flora and the abundance of fresh grass in the Trabzon regions 22 where these butters are produced [3]. The comparison of traditional and commercial butters based on their fatty acid composition reveals some differences. Traditional butters have a slightly higher total of short and medium saturated fatty acids (11.81 %) compared to commercial butters (10.90 %). For individual fatty acids, traditional butters contain more butyric acid (2.54 % vs. 2.40 %) and caproic acid (1.95 % vs. 1.72 %), while commercial butters have slightly more capric acid (2.53 % vs. 2.90 %) and caprylic acid (1.10 % vs. 1.27 %). Both types have similar amounts of lauric acid (3.06 %). In terms of long chain fatty acids, traditional butters have higher concentrations of myristic acid (12.44 % vs. 12.14 %) and palmitic acid (33.88 % vs. 34.30 %). The data suggests that while the types of fatty acids present are largely consistent, traditional butters tend to have marginally higher concentrations of certain saturated fatty acids compared to commercial butters. It can be many reasons for diversity of fatty acids in our study. For example; the diet and feed of dairy cows, which affect the milk's fatty acid content based on whether cows graze on fresh grass or stored feeds, play a significant role [40]. Production methods impact fatty acid profiles, with traditional methods retaining a broader spectrum of fatty acids due to minimal processing, while commercial methods involve processes like homogenization and pasteurization [44]. The use of starter cultures in butter production can modify the fatty acid profile through bacterial metabolic activities [32].

**Table 5:** Fatty acid composition of butters.

	Samples^a^											
Fatty acids	6	8	16	17	18	19	20	23	Mean	26	27	Mean
Short and medium saturated fatty acids												
Butyric Acid	2.56	2.03	2.28	2.35	2.31	3.13	3.01	2.63	**2.54**	2.33	2.47	2.40
Caproic Acid	2.03	1.76	1.76	1.83	1.69	2.32	2.45	1.76	**1.95**	1.69	1.74	1.72
Caprylic Acid	1.22	1.34	1.16	1.2	1.08	1.47	1.61	1.11	**1.27**	1.1	1.1	1.10
Capric Acid	2.81	3.42	2.55	2.85	2.46	3.23	3.45	2.45	**2.90**	2.57	2.48	2.53
Lauric Acid	3.25	4.46	2.95	3.27	2.92	0	4.68	2.92	**3.06**	3.13	2.99	3.06
Tridecanoic Acid	0.08	0.11	0.08	0.07	0.08	0.09	0.13	0.08	**0.09**	0.1	0.09	0.10
Total	11.95	13.12	10.78	11.57	10.54	13.69	11.88	10.95	**11.81**	10.92	10.87	10.90
Long chain fatty acids												
Myristic Acid	11.6	14.08	11.36	11.96	10.78	13.98	15.28	10.48	**12.44**	11.19	11.04	12.14
Pentadecanoic Acid	1.15	1.67	1.14	1.33	1.25	1.69	1.45	1.21	**1.36**	1.16	1.18	1.33
Palmitic Acid	35.25	31.55	26.11	32.34	29.76	43.18	44.25	28.63	**33.88**	32.07	30.27	34.30
Margaric Acid	0.67	0.8	0.74	0.74	0.65	1.01	0.71	0.86	**0.77**	0.59	0.59	0.74
Stearic Acid	11.61	6.86	12.62	11.88	11.79	17.35	14.41	12.9	**12.43**	9.78	10.41	12.62
Total	60.28	54.96	51.97	58.25	54.23	77.21	76.1	54.08	**60.89**	54.79	53.49	61.13
Monounsaturated fatty acids												
Palmitoleic Acid	1.85	1.83	1.53	1.5	1.74	1.98	1.9	0.06	**1.55**	1.89	1.88	1.56
Oleic Acid	19.97	19.55	26.5	21.22	24.27	29.8	26.45	25.88	**24.21**	24.79	26.05	25.33
Total	21.82	21.38	28.03	22.72	26.01	31.78	28.35	25.94	**25.75**	26.68	27.93	26.90
Polyunsaturated fatty acids												
Linoleic Acid	2.01	1.63	1.89	1.89	2.06	2.62	3.04	2.21	**2.17**	2.56	2.4	2.37
Linolenic Acid	0.43	1.06	0.71	0.7	0.65	0.78	0.34	0.72	**0.67**	0.24	0.31	0.55
Linolelaidic Acid	0.51	0.43	1.06	0.07	1.02	0.84	0.89	0.64	**0.68**	0.8	1.08	0.75
Eicosanoic Acid	0.25	0.18	0.25	0.26	0.22	0.29	0.25	0.27	**0.25**	0.18	0.18	0.24
Total	3.2	3.3	3.91	2.92	3.95	4.53	4.52	3.84	**3.77**	3.78	3.97	3.91
Grand total	182.55	172.4	178.6	179.35	178.92	240.73	229.82	178.67	**192.63**	181.42	181.65	195.40

a 6.8.16.17.18.19.20.23–23: Traditonal butters; 26.27: Commercial butters.

Compared to the study by Ozcan et al. (2016), which found palmitic acid as dominant and oleic acid as the major monounsaturated fatty acid in Trabzon butter, these results are consistent. The butter samples also had low n-6/n-3 ratios and low atherogenic and thrombogenic indexes, aligning with findings from other studies in the Azores and by Méndez-Cid et al. [34]. The content of butyric, oleic, and linoleic acids in the samples also falls within the ranges reported by Middaugh et al. [36] and Çavdar [12], with a saturated to unsaturated fatty acids ratio similar to those observed in market butters.

### 3.6 Volatile aroma components

The volatile compounds in butter are identified in Table 6. As seen from table, traditional butters exhibit a higher diversity of volatiles, with prominent compounds such as butanoic acid (0.91–16.02 %), hexanoic acid (1.82–46.59 %), and octanoic acid (20.28–43.37 %), which contribute to the rich and complex aroma profiles. Traditional butters also contain butanoic acid nonyl ester (0.33–4.53 %) and n-butanoic acid 2-ethylhexyl ester (0.54–0.92 %), adding fruity and sweet notes. In contrast, commercial butters have lower concentrations of these acids, with hexanoic acid ranging from 26.24 % to 46.59 % and octanoic acid between 20.07 % and 25.28 %. Additionally, nonanal, contributing a sweet, fatty odor, is present in commercial butters but absent in traditional ones. The presence of mechanical churning and pre-acidified creams in commercial butter production likely leads to lower butanoic acid levels (6.96–8.55 %) compared to traditional butters (0.91–16.02 %). Interestingly, commercial butters have higher levels of octanoic acid ethyl ester (1.18–2.33 %), enhancing their fruity aroma. These differences highlight how traditional production methods and freshness contribute to the richer and more varied aroma profiles in traditional butters, while commercial butters, due to standardized processes and ingredients, exhibit more consistent but less complex aromas. Additionally, limonene (0.45–2.66 %) and indene, although present in lower concentrations, contribute citrusy and musty notes, respectively. This detailed comparison emphasizes the impact of production techniques and ingredients on the volatile profiles of butters. Şengül et al. [54] noted lower amounts of butanoic acid and hexanoic acid in butter produced without the addition of starter culture compared to those produced with starter culture. The results of their study differ from ours regarding these two values. This discrepancy is believed to stem from traditional butter being fresher, while creams used in the production of commercial butter are assumed to be pre-acidified, thus waiting longer [21]. Additionally, mechanical churning applied to butter produced in commercial settings is also considered effective. Indeed, Cadwallader and Singh [9] reported that whipping cream could increase the concentrations of oxidation products and improve taste if not excessive. Our findings in Table 6 supports this by showing that the overall acceptability scores of commercial butter are higher than those of traditional ones. One of the interesting findings of our study was the detection of diethyl phthalate in 2 traditional butter samples, suggesting possible contamination from plastic containers used during churning. Phthalates are materials added to plastic polymers to enhance flexibility, and their presence in dairy products has been identified in recent years. Moreover, due to their property of being soluble in fat rather than water, they are more prevalent in high-fat products such as butter [16]. Our findings showed that components such as N-butanoic acid 2-ethylhexyl ester, decanoic acid, pentadecane, dodecanoic acid, 1-methyl ester, tetradecanoic acid, *cis*-11-hexadecanal, hexanoic acid, ethyl ester, tetradecanoic acid, ethyl ester, 2-hexadecene, 2,6,10,14-tetramethyl-, n-hexadecenoic acid, E−11-hexadecenoic acid, ethyl ester, 9-octadecenoic acid, ethyl ester were found only in some traditionally produced butters while not detected in commercial butters. The quantities of octanoic and n-decanoic acids were higher in traditional butters ranging from 13.36 to 43.37 % and 17.53–27.44 % respectively, whereas in commercial butters, they showed variations between 20.07-25.28 % and 8.19–12.11 % respectively.

**Table 6:** Volatile aroma compounds of butters.

				Samples*											Mean
	RT^a^	CI^b^	LCI^c^	6	8	16	17	18	19	20	23	Mean	26	27	
3-Buten-2-ol-3-Methyl	4.497	665	673	ND	ND	ND	0.02	ND	ND	ND	ND	**122.05**	ND	ND	**0.00**
Butanoic acid	8.91	821	821	12.09	2.05	0.91	4.79	16.02	9.57	ND	7.99	**154.94**	6.96	8.55	**7.76**
2-Heptanone	11.385	891	891	ND	1.94	ND	0.1	ND	ND	ND	ND	**163.22**	7.59	2.59	**5.09**
Benzaldehyde	13.523	963	963	ND	0.15	ND	ND	ND	ND	ND	ND	**176.33**	0	0	**0.00**
Hexanoic acid	14.906	1013	1013	14.16	1.82	25.98	27.33	23.22	16.44	5.38	21.67	**197.90**	26.24	46.59	**36.42**
Limonene	15.365	1032	1032	0.45	2.5	1.27	ND	ND	2.08	ND	0.42	**189.64**	1.57	2.66	**2.12**
Indene	15.768	1049	1049	ND	ND	ND	ND	ND	ND	ND	0.43	**192.20**	0	0	**0.00**
Heptanoic acid	16.557	1082	1083	ND	0.5	ND	0.26	2.67	ND	ND	ND	**198.64**	0.71	0.39	**0.55**
2-Nonanone	16.813	1093	1093	ND	2.18	ND	0.47	ND	ND	ND	0.25	**200.52**	8.65	2.49	**5.57**
Nonanal	17.082	1104	1104	ND	0.05	ND	ND	ND	ND	ND	ND	**202.28**	0.98	0.42	**0.70**
2-Phenylethanol	17.355	1117	1117	ND	1.16	0.72	ND	ND	ND	ND	ND	**204.84**	0.77	1.02	**0.90**
Octanoic acid	18.803	1186	1186	28.1	44.37	25.58	35.82	20.28	43.37	13.36	31.25	**239.36**	25.28	20.07	**22.68**
Octanoic acid. ethyl ester	18.963	1194	1194	ND	ND	ND	ND	ND	1.18	2.33	0	**219.13**	ND	ND	**0.00**
Dodecane	19.024	1197	1200	ND	ND	ND	ND	ND	ND	ND	ND	**219.64**	ND	0.65	**0.33**
Decanal	19.172	1204	1204	ND	0.24	ND	ND	ND	ND	ND	ND	**220.67**	ND	0.31	**0.16**
Acetic acid. 2-phenylethyl ester	20.208	1259	1258	ND	1.02	ND	ND	ND	ND	ND	ND	**230.75**	ND	ND	**0.00**
Nonanoic acid	20.429	1271	1271	0.14	1.49	0.85	0.36	ND	6.4	12.57	0.15	**234.94**	ND	0.64	**0.32**
2-Undecanone	20.676	1284	1284	8.14	0.43	0.78	ND	ND	ND	ND	0.6	**236.24**	ND	0.46	**0.23**
Isopentyl 3-hydroxy-2-methylenebutanoate	20.771	1289	1284.2	0.77	2.67	0.56	0.83	4.2	ND	ND	ND	**236.64**	ND	0.61	**0.31**
n-Butyric acid 2-ethylhexyl ester	21.101	1306	1314	0.74	0.54	ND	ND	ND	ND	0.92	ND	**240.30**	ND	ND	**0.00**
Butanoic acid. 2-methyl-. heptyl ester	21.218	1319	1317	0.61	0.24	0.8	0.22	4.35	ND	ND	0.94	**242.22**	0.62	0.93	**0.78**
n-Decanoic acid	22.427	1382	1382	27.44	19.48	26.47	22.29	17.63	17.53	19.59	25.36	**269.29**	12.11	8.19	**10.15**
Decanoic acid	22.558	1389	1389	ND	7.59	ND	1.04	ND	ND	ND	0.4	**255.42**	ND	ND	**0.00**
3-(4-Isopropylphenyl)-2-methylpropanal	23.663	1457	1436.5	0.33	0.31	ND	ND	ND	ND	ND	ND	**265.25**	ND	ND	**0.00**
n-Heptyl hexanoate	23.997	1477	1482	0.38	0.39	0.95	0.18	2.28	ND	0.97	2.34	**271.86**	0.37	ND	**0.19**
2-Tridecanone	24.218	1491	1491	ND	1.37	ND	ND	ND	ND	ND	ND	**273.42**	0.81	ND	**0.41**
Pentadecane	24.231	1492	1500	ND	ND	ND	ND	ND	ND	ND	0.49	**274.25**	ND	ND	**0.00**
Butanoic acid. nonyl ester	24.378	1501	1489	0.81	1.88	5.1	0.41	4.78	3.44	1.69	2.82	**275.94**	4.53	0.33	**2.43**

*6.8.16.17.18.19.20.23–23: Traditonal butters; 26.27: Commercial butters.

a : Retention time.

b : Covax index.

c : Literature covax index; ND: Not Detected.

## 4 Conclusion

Traditional butters have higher moisture content and lower fat percentages compared to commercial ones. Traditional butters exhibit slightly higher acidity and peroxide values. The color analysis shows that traditional butters have a wider range of color values. Overall, traditional butters display greater variability in their physicochemical properties, while commercial butters offer more consistency and stability. On the other hand, traditional butters generally have lower cholesterol except one sample and higher vitamin contents. The fatty acid profiles in traditional butters are more diverse, with higher levels of beneficial fatty acids like oleic acid. Additionally, traditional butters contain a broader range of volatile compounds. In contrast, commercial butters show more consistent but less diverse profiles, with significant levels of oleic and palmitic acids.

## Data and code availability

Data will be made available on request.

## CRediT authorship contribution statement

**Engin Gündoğdu:** Writing – review & editing, Writing – original draft, Visualization, Validation, Supervision, Resources, Methodology, Investigation, Formal analysis, Data curation, Conceptualization. **Musa Beşer:** Investigation, Formal analysis, Data curation.

## Declaration of competing interest

The authors declare that they have no known competing financial interests or personal relationships that could have appeared to influence the work reported in this paper.

## References

[1] Agyare A.N., Liang Q., Song X., Zhang Y., Yang J., Shi Y. (2022). Oxidative stability and sensory evaluation of sodium caseinate-based yak butter powder. Sci. Rep..

[2] Akgül H.İ., Şengül M. (2019).

[3] Akgül H.İ., Şengül M., Ürkek B., Kotan T.E. (2021). Determination of physicochemical and microbiological properties and fatty acid composition of butter produced in Trabzon, Turkey. Acta Sci. Technol..

[4] Akgül H.İ., Şengül M., Ürkek B., Kotan T.E. (2021). Determination of physicochemical and microbiological properties and fatty acid composition of butter produced in Trabzon, Turkey. Acta Sci. Technol..

[5] Aloğlu H., Öner Z. (2006). Assimilation of cholesterol in broth, cream, and butter by probiotic bacteria. Eur. J. Lipid Sci. Technol..

[6] Altun I., Andic S., Tuncturk Y., Cecen A., Findik O. (2011). Some chemical characteristics of butters obtained from Van market. Kafkas Üniversitesi Veteriner Fakültesi Dergisi..

[7] Ayaşan T., Karakozak E. (2010).

[8] Bayır A.G., Bilgin M.G., Özkan B., İtmez M., Atacan B., Yavuz B., Yormaz H.İ. (2022). Evaluation of physico-chemical properties according to TS 1331 of butters sld in Istanbul, Turkey. KASAV International Journal of Health Sciences.

[9] Cadwallader K.R., Singh T. (2009). Flavours and off-flavours in milk and dairy products. Advanced dairy chemistry: Volume 3: Lactose, water, salts and minor constituents.

[10] Cheng S., Wu S. (2024). A shelf life prediction method for butter based on the effects of β‐carotene on colour and oxidative stability. Int. J. Dairy Technol..

[11] Cheng Z., O'Sullivan M.G., Miao S., Kerry J.P., Kilcawley K.N. (2022). Sensorial, cultural and volatile properties of milk, dairy powders, yoghurt and butter: a review. Int. J. Dairy Technol..

[12] Çavdar H.K. (2022). Assessment of physicochemical characteristics, oxidative, and thermal properties of butters. GIDA/The Journal of Food..

[13] Çolakoğlu G., Öztürk H.Ö. (2018). Bursa ve Samsun illerindeki tereyağlardan izole edilen funguslar üzerine araştırmalar. Mantar Dergisi.

[14] Delgado A., Al-Hamimi S., Ramadan M.F., Wit M.D., Durazzo A., Nyam K.L., Issaoui M. (2020). Contribution of tocols to food sensorial properties, stability, and overall quality. J. Food Qual..

[15] Dudkiewicz A., Hayes W., Onarinde B. (2022). Sensory quality and shelf-life of locally produced British butters compared to large-scale, industrially produced butters. Br. Food J..

[16] Fierens T., Van Holderbeke M., Willems H., De Henauw S., Sioen I. (2013). Transfer of eight phthalates through the milk chain — a case study. Environ. Int..

[17] Fındık O., Andiç S. (2017). Some chemical and microbiological properties of the butter and the butter oil produced from the same raw material. Lwt.

[18] Fletouris D., Botsoglou N., Psomas I., Mantis A. (1998). Rapid determination of cholesterol in milk and milk products by direct saponification and capillary gas chromatography. J. Dairy Sci..

[19] Ghasemloy Incheh K., Hassanzadazar H., Forouzan S., Mozafarian E., Aminzare M., Hashemi M. (2017). A survey on the quality of traditional butters produced in West Azerbaijan province, Iran. Int. Food Res. J..

[20] Hassanzad Azar H., Salim A., Forghani M., Aminzare M. (2017). Chemical quality and fatty acid profile of zanjan traditional butter. Annual Research & Review in Biology.

[21] Jacobsen D., Evans T.A. (1941).

[22] Jensen R.G., Ferris A.M., Lammi-Keefe C.J. (1991). The composition of milk fat. J. Dairy Sci..

[23] Kalac P. (2012). Carotenoids, ergosterol and tocopherols in fresh and preserved herbage and their transfer to bovine milk fat and adipose tissues: a review. Journal of Agrobiology..

[24] Kashaninejad M., Razavi S.M., Mazaheri Tehrani M., Kashaninejad M. (2017). Effect of extrusion conditions and storage temperature on texture, colour and acidity of butter. Int. J. Dairy Technol..

[25] Khan I.T., Bule M., Ullah R., Nadeem M., Asif S., Niaz K. (2019). The antioxidant components of milk and their role in processing, ripening, and storage: functional food. Vet. World.

[26] Khaskheli G.B., Khaskheli A.A., Magsi A.S., Barham G.S., Jamali M.A., Khaskheli A.A. (2020). Study on quality characteristics of sweet and sour cream butter: quality assessment of butter, Proceedings of the Pakistan Academy of Sciences: B. Life and Environmental Sciences.

[27] Kisza J., Staniewski B., Juskiewicz M. (1996). The content of cholesterol in butter depending on the season and production method. Pol. J. Food Nutr. Sci..

[28] Kurt A., Çakmakçı S., Çağlar A. (2015).

[29] Lal D., Sharma V., Seth R. (2019).

[30] Li W., Li X., Wu Z., Li X., Yang A., Tong P., Chen H. (2016). Quantitative determination of β-carotene aided by hexane/ethanol extraction. J. Chem. Soc. Pakistan.

[31] Lordan R., Zabetakis I. (2017). Invited review: the anti-inflammatory properties of dairy lipids. J. Dairy Sci..

[32] Mallia S., Escher F., Schlichtherle-Cerny H. (2008). Aroma-active compounds of butter: a review. Eur. Food Res. Technol..

[33] Martin B., Verdier-Metz I., Buchin S., Hurtaud C., Coulon J.-B. (2005). How do the nature of forages and pasture diversity influence the sensory quality of dairy livestock products?. Anim. Sci..

[34] Méndez-Cid F.J., Centeno J.A., Martínez S., Carballo J. (2017). Changes in the chemical and physical characteristics of cow's milk butter during storage: effects of temperature and addition of salt. J. Food Compos. Anal..

[35] Metin M. (2009). Ege Meslek Yüksekokulu Yayınları, İzmir.

[36] Middaugh R.P., Baer R., Casper D., Schingoethe D., Seas S. (1988). Characteristics of milk and butter from cows fed sunflower seeds. J. Dairy Sci..

[37] Milovanovic B., Djekic I., Miocinovic J., Djordjevic V., Lorenzo J.M., Barba F.J., Mörlein D., Tomasevic I. (2020). What is the color of milk and dairy products and how is it measured?. Foods.

[38] Milovanovic B., Tomovic V., Djekic I., Miocinovic J., Solowiej B.G., Lorenzo J.M., Barba F.J., Tomasevic I. (2021). Colour assessment of milk and milk products using computer vision system and colorimeter. Int. Dairy J..

[39] Mohamad R., Agus B.A.P., Hussain N. (2019). Changes of phytosterols, rheology, antioxidant activity and emulsion stability of salad dressing with cocoa butter during storage. Food Technol. Biotechnol..

[40] O'Callaghan T.F., Mannion D., Apopei D., McCarthy N.A., Hogan S.A., Kilcawley K.N., Egan M. (2019). Influence of supplemental feed choice for pasture-based cows on the fatty acid and volatile profile of milk. Foods.

[41] Ostadzadeh M., Habibi Najafi M.B., Ehsani M.R. (2023). Lactic acid bacteria isolated from traditional Iranian butter with probiotic and cholesterol-lowering properties: in vitro and in situ activity. Food Sci. Nutr..

[42] Ozcan T., Akpinar-Bayizit A., Yilmaz-Ersan L., Cetin K., Delikanli B. (2016). Evaluation of fatty acid profile of Trabzon butter. International Journal of Chemical Engineering and Applications.

[43] Pădureţ S. (2021). The effect of fat content and fatty acids composition on color and textural properties of butter. Molecules.

[44] Qin C., Liu L., Wang Y., Leng T., Zhu M., Gan B., Xie J., Yu Q., Chen Y. (2022). Advancement of omics techniques for chemical profile analysis and authentication of milk. Trends Food Sci. Technol..

[45] Rutkowska J., Adamska A. (2011). Fatty acid composition of butter originated from North-Eastern region of Poland. Pol. J. Food Nutr. Sci..

[46] Saeen Z.S., Khani M., Noghani V.F. (2022). Effects of chia seed extract on chemical and sensory properties of sweet cream butter during refrigerated storage. Iranian Food Science & Technology Research Journal.

[47] Schiavon S., Paolini M., Guzzon R., Mancini A., Larcher R., Villegas T.R., Franciosi E. (2021). Bacterial complexity of traditional mountain butter is affected by the malga-farm of production. Microorganisms.

[48] Seçkin A.K., Gursoy O., Kinik O., Akbulut N. (2005). Conjugated linoleic acid (CLA) concentration, fatty acid composition and cholesterol content of some Turkish dairy products. LWT--Food Sci. Technol..

[49] Seçkin A.K., Metin M. (2011). Süt ve ürünlerinde kolesterol oksidasyon ürünleri. Pamukkale Üniversitesi Mühendislik Bilimleri Dergisi.

[50] Sevmiş S., Andiç Ş. (2020).

[51] Shekhara Naik R., Pavithra R., Prakruthi M. (2020).

[52] Silva C.C.G., Silva S.P.M., Prates J.A.M., Bessa R.J.B., Rosa H.J.D., Rego O.A. (2019). Physicochemical traits and sensory quality of commercial butter produced in the Azores. Int. Dairy J..

[53] Staniewski B., Ogrodowska D., Staniewska K., Kowalik J. (2021). The effect of triacylglycerol and fatty acid composition on the rheological properties of butter. Int. Dairy J..

[54] Şengül M., Akgül H., Ürkek B. (2023). The aroma profile of butters produced using different starter cultures. Research Square.

[55] Vioque-Amor M., Gómez-Díaz R., Del Río-Celestino M., Avilés-Ramírez C. (2023). Butter from different species: composition and quality parameters of products commercialized in the south of Spain. Animals.

